# Fusobacterium naviforme Liver Abscess Secondary to a Non-Meckelian Ileal Diverticulum Successfully Treated by Laparoscopic Resection After Failed Conservative Therapy

**DOI:** 10.7759/cureus.91772

**Published:** 2025-09-07

**Authors:** Kenta Mitsusada, Takashi Hamano, Yasuyuki Kobayashi

**Affiliations:** 1 Department of Colorectal Surgery, Seirei Hamamatsu General Hospital, Hamamatsu, JPN

**Keywords:** fusobacterium naviforme, ileal diverticulum, laparoscopic ileocecal resection, pyogenic liver abscess, source control

## Abstract

Pyogenic liver abscess (PLA) is most commonly caused by Klebsiella pneumoniae or Escherichia coli of biliary or intestinal origin. Fusobacterium species are rare pathogens, and when identified, their possible sources include not only oral or pharyngeal lesions but also lesions of the lower gastrointestinal tract. We report the case of A 77-year-old female who presented with fever and anorexia. Laboratory findings showed an elevated white blood cell count and C-reactive protein (CRP). Contrast-enhanced CT revealed multiple liver abscesses in the right lobe and an abscess dorsal to the terminal ileum. Pus from ultrasound-guided drainage yielded Fusobacterium naviforme (F. naviforme). Despite carbapenem therapy and drainage, the infection persisted. Based on imaging findings suggestive of an ileal diverticulum as the source, laparoscopic ileocecal resection was performed. Pathology confirmed continuity between the diverticulum and a subserosal abscess cavity. Following surgery, the liver abscesses regressed markedly, and the patient was discharged on postoperative day 12.

When Fusobacterium species are isolated from PLA, evaluation for lower gastrointestinal lesions should definitely be considered. Small abscesses (less than 3 cm) may resolve with antibiotics alone, but larger abscesses generally require drainage, and surgical intervention is indicated when conservative therapy fails or a persistent source is identified. To our knowledge, this is the first reported case of F. naviforme PLA arising from a non-Meckelian ileal diverticulum, successfully treated by surgery after the failure of conservative therapy.

## Introduction

Pyogenic liver abscess (PLA) is a rare but clinically significant disease, with an estimated incidence of 1.1-3.6 cases per 100,000 population per year [1,2]. Its clinical presentation is often nonspecific, including fever, abdominal pain, and malaise, which can delay diagnosis. Advances in imaging and microbiological techniques have enhanced detection, and modern therapies have significantly reduced mortality rates compared to historical averages of 60-80%. However, recent studies have reported inpatient mortality between approximately 6 and 10% [2,3], underscoring its potential severity. The etiology of PLA varies geographically: in East Asia, Klebsiella pneumoniae is the most common pathogen, whereas Escherichia coli predominates in Western countries [2]. Diabetes mellitus is a well-established risk factor for PLA, particularly in Klebsiella infections, and was present in this patient. Fusobacterium species, although rare, have been increasingly recognized as causative organisms. These anaerobic bacteria are typically associated with oral or pharyngeal infections but can also arise from lower gastrointestinal lesions, including appendicitis, Meckel’s diverticulum [4,5].

The management of PLA depends on the abscess size, number, and the ability to achieve source control. Small abscesses (less than 3 cm) may resolve with antibiotics alone, while larger ones often require percutaneous drainage, and refractory cases may necessitate surgery [6,7]. Identifying and addressing the underlying source of infection is crucial, as persistent foci can result in recurrent or non-resolving abscesses. We present a case of Fusobacterium naviforme (F. naviforme) PLA originating from a non-Meckelian (i.e., acquired) ileal diverticulum in a female patient. Her condition failed to improve with conservative therapy (antibiotic therapy and percutaneous drainage), but she was successfully cured by laparoscopic ileocecal resection, illustrating the importance of surgical source control in selected cases.

## Case presentation

A female in her late 70s with a history of hypertension, dyslipidemia, and diabetes presented with a one-week history of fever and anorexia. She had no history of prior dental treatment. On physical examination, she was febrile (38.7 °C) with right lower quadrant abdominal tenderness but no peritoneal signs. Her BMI was 20.8 kg/m², HbA1c was 7.2%, and she was a never-smoker.

Laboratory tests

The detailed results are summarized in Table 1. The patient demonstrated marked leukocytosis with neutrophil predominance and a markedly elevated C-reactive protein (CRP) level, along with mild hypokalemia. Liver and renal function tests were within normal limits.

**Table 1:** Laboratory findings on admission

Parameter	Patient value	Reference range
Sodium (Na)	138 mEq/L	135 – 145 mEq/L
Potassium (K)	3.2 mEq/L	3.5 – 5.0 mEq/L
Chloride (Cl)	99 mEq/L	98 – 106 mEq/L
Blood urea nitrogen (BUN)	14 mg/dL	8 – 20 mg/dL
Creatinine	0.59 mg/dL	0.6 – 1.2 mg/dL
White blood cell count	18,720/μL	4,000 – 10,000/μL
Neutrophils	86%	40 – 70%
C-reactive protein (CRP)	26.49 mg/dL	<0.3 mg/dL
Aspartate aminotransferase (AST)	22 U/L	10 – 40 U/L
Alanine aminotransferase (ALT)	22 U/L	7 – 56 U/L

Imaging

**Figure 1:**
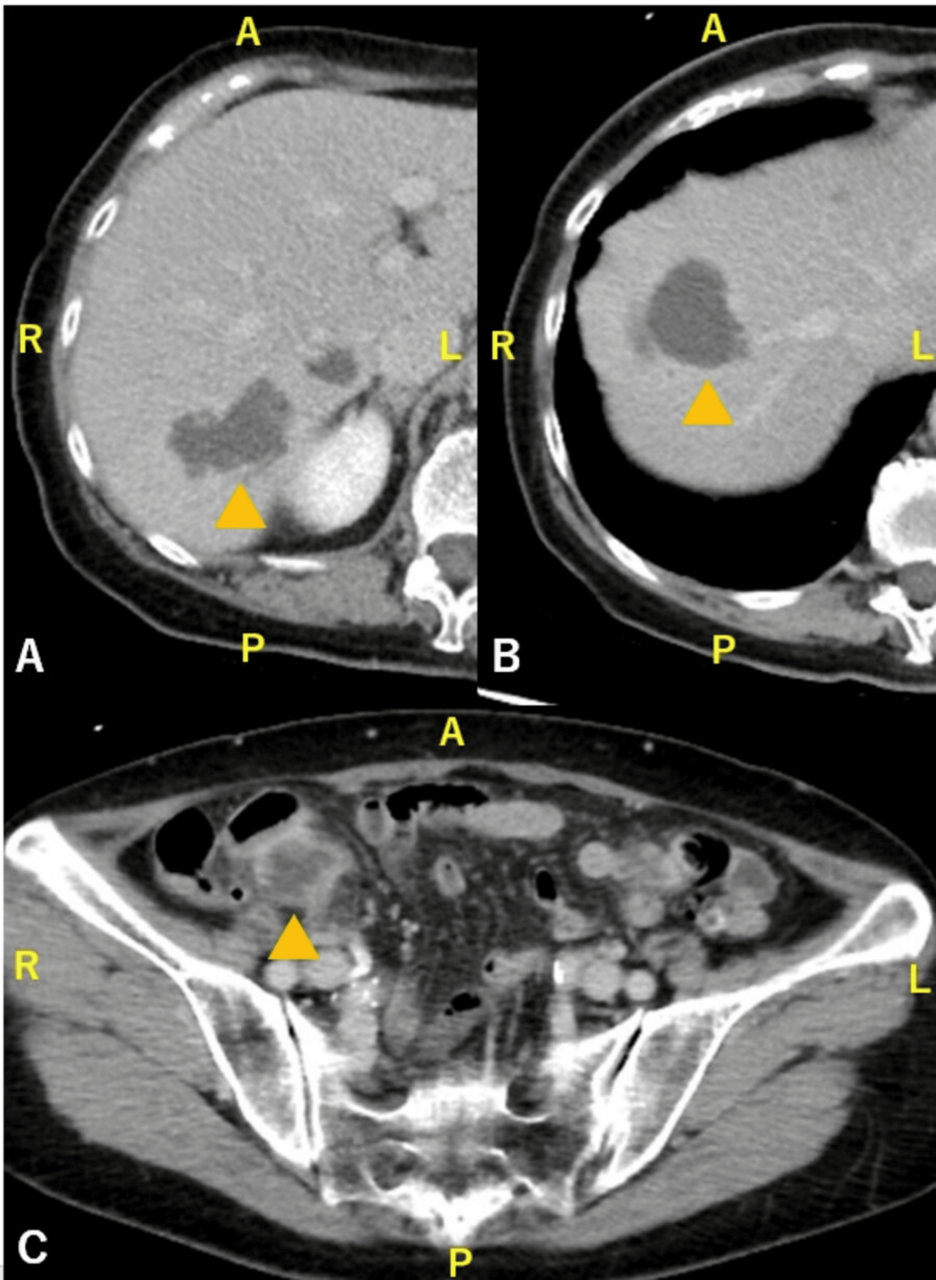
Contrast-enhanced CT at admission (A, B) Multiple abscesses are seen in the right hepatic lobe (arrowheads). (C) An additional abscess is noted dorsal to the terminal ileum (arrowhead) CT: computed tomography

Contrast-enhanced CT revealed multiple abscesses in the right hepatic lobe (measuring 2.9 cm in maximum diameter) and an abscess adjacent to the terminal ileum (Figure 1).

Treatment course

**Figure 2:**
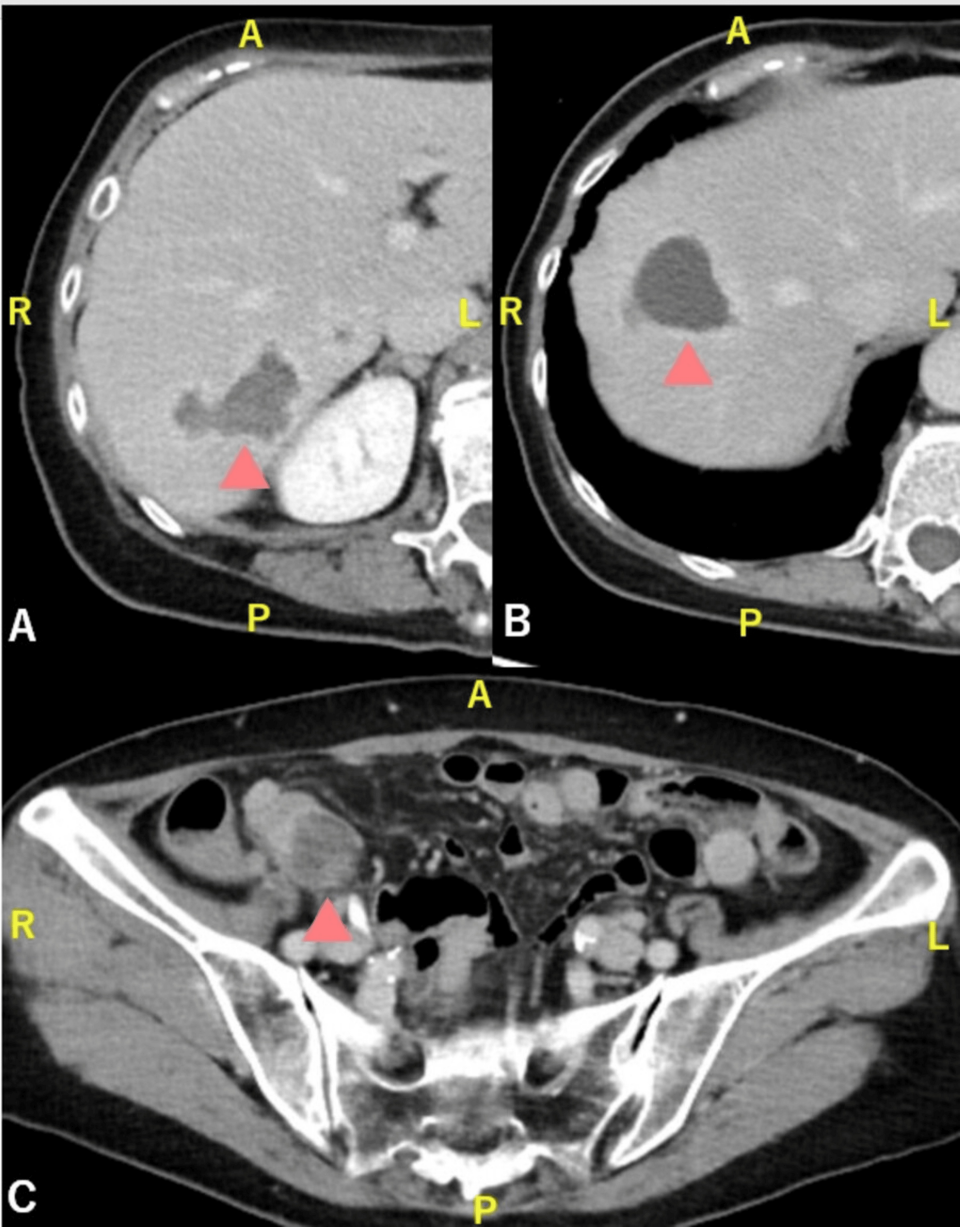
Follow-up contrast-enhanced CT after meropenem therapy and ultrasound-guided drainage of the hepatic abscesses (A, B) No remarkable improvement is observed in the liver abscesses (arrowheads). (C) The abscess adjacent to the terminal ileum persists (arrowhead) CT: computed tomography

Ultrasound-guided aspiration drainage of the largest hepatic abscess was performed with two puncture sites, yielding a total of 17 mL of purulent material; no catheter was left in place. Culture of the pus grew F. naviforme, identified by anaerobic culture and matrix-assisted laser desorption-ionization time of flight (MALDI-TOF) mass spectrometry. Antimicrobial susceptibility testing showed the isolate was susceptible to carbapenems. The patient was treated with intravenous meropenem 1 g every eight hours for 12 days, but follow-up CT demonstrated poor improvement (Figure 2).

**Figure 3:**
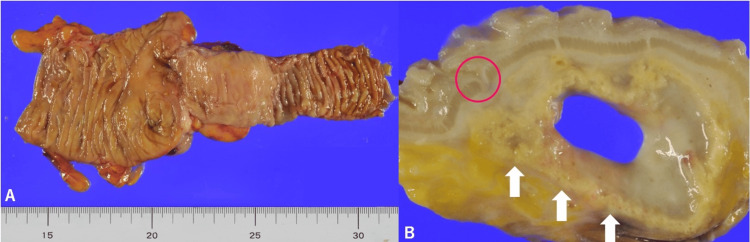
Gross findings of the resected specimen (A) The ileocecal resection specimen, including the terminal ileum and cecum. (B) Cross-section of the terminal ileum reveals a 30-mm subserosal cavity with degenerative change of adipose tissue (white arrows). A diverticulum continuous from the ileal mucosa to the abscess cavity is also identified (red circle)

**Figure 4:**
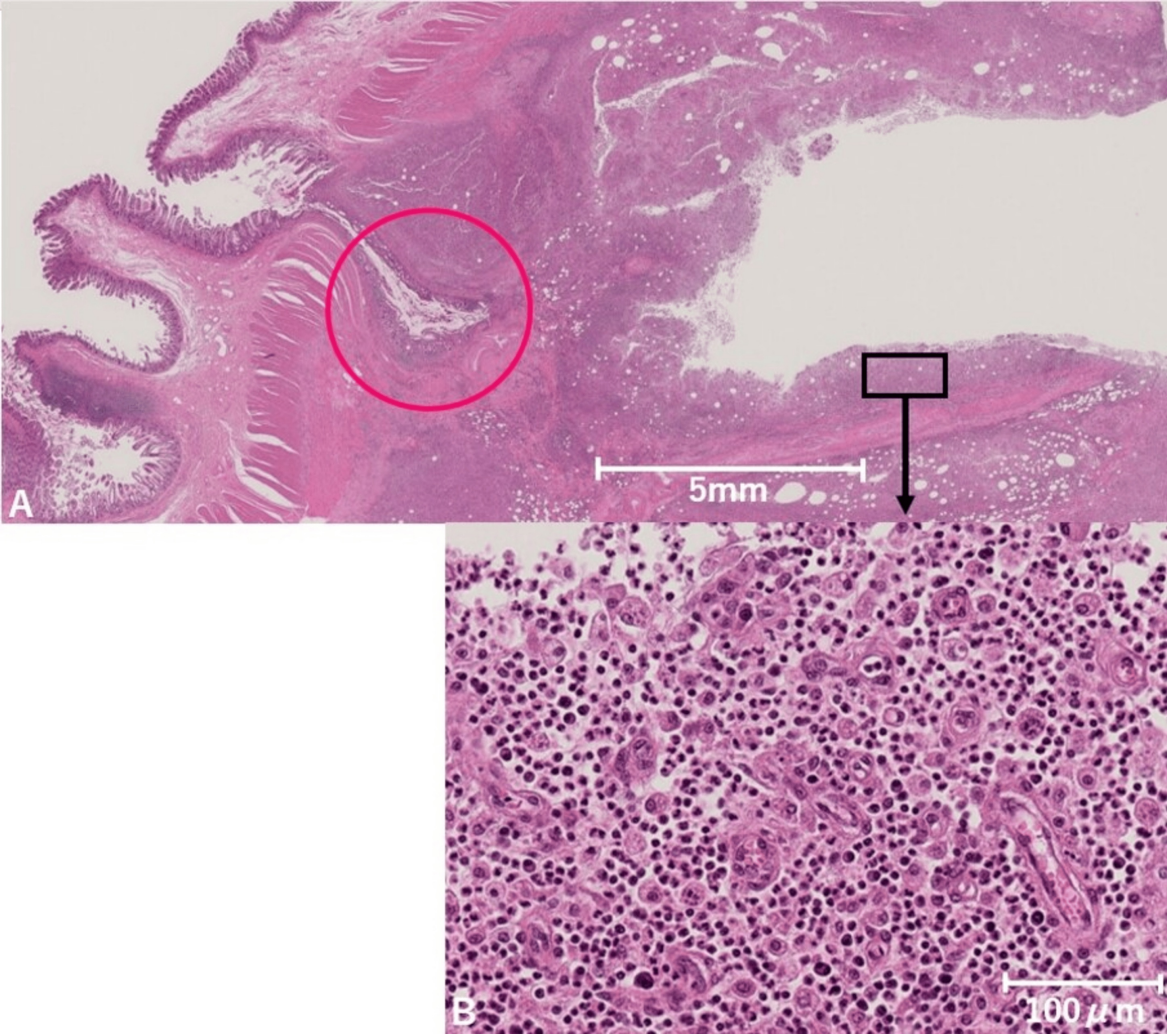
Histopathological findings of the ileal diverticulum (A) Hematoxylin and eosin (H&E) staining (at 0.5× magnification with scale bar = 5 mm) shows an ileal diverticulum (red circle) with marked neutrophilic infiltration and abscess formation extending from the deep portion. (B) Higher magnification (20×, scale bar = 100 µm) demonstrates dense neutrophilic infiltration

Because of a persistent infection suspected to originate from an ileal diverticulum, surgical resection of the source was planned. Intraoperatively, the terminal ileum was found to be mildly adherent to the mesentery and appendix. Since the abscess cavity was not clearly visible in the operative field, laparoscopic ileocecal resection was performed to ensure complete removal of the infectious source. The gross specimen is shown in Figure 3. Pathology demonstrated continuity between the diverticulum and a subserosal abscess cavity (Figure 4).

Outcome

**Figure 5:**
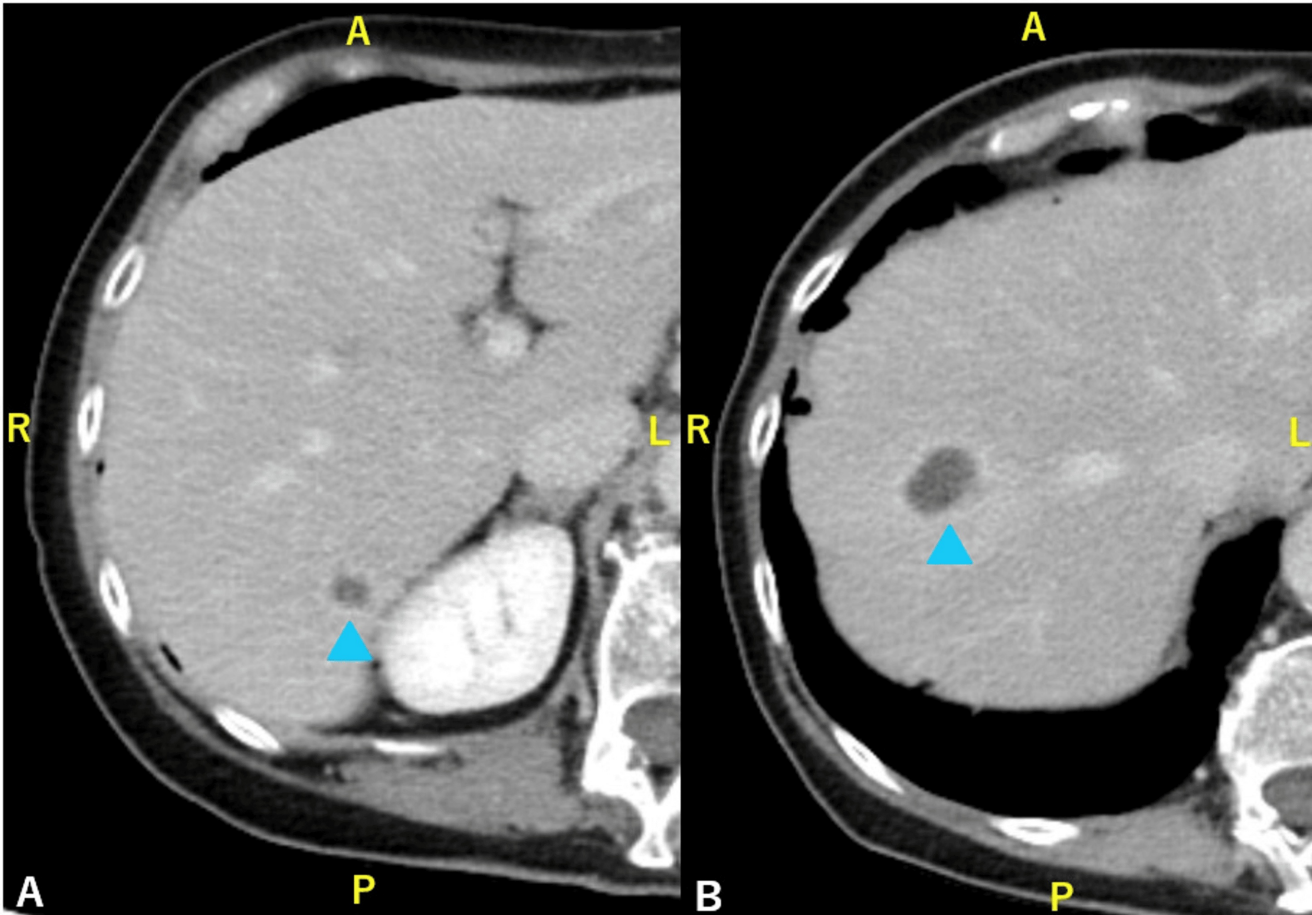
Postoperative contrast-enhanced CT on day six after laparoscopic ileocecal resection (A, B) Marked regression of the hepatic abscesses is evident (arrowheads) CT: computed tomography

Postoperative CT on day six revealed marked regression of the liver abscesses (Figure 5). The patient was discharged on postoperative day 12.

## Discussion

Fusobacterium and lower gastrointestinal sources

Fusobacterium species are rare but established pathogens linked to PLA [1,2]. Although oral and pharyngeal origins are more common, lower gastrointestinal lesions - including diverticulitis, appendicitis, and Meckel’s diverticulum - have also been reported [4,5]. In our patient, a non-Meckelian ileal diverticulum was identified as the persistent source.

Therapeutic considerations

The treatment of PLA depends on the abscess size and clinical course. Small abscesses (less than 3 cm) may resolve with antibiotics alone, but larger abscesses usually require percutaneous drainage [6,7]. When conservative treatment fails or a continuous source of infection exists, surgical intervention is warranted for definitive source control [1,6]. In fact, several reports have described cases of Fusobacterium-related PLA requiring resection of gastrointestinal lesions. For example, in a case where Meckel’s diverticulum was identified as the source of infection, surgical resection of the diverticulum was needed for cure [5]. Another report described inflammatory adhesions involving the rectosigmoid colon and appendix as the infectious source, where laparoscopic adhesiolysis and appendectomy were ultimately required to achieve resolution [8].

These reports indicate that when gastrointestinal sources persist, conservative therapy alone is insufficient, and surgical treatment is essential to achieve a definitive cure and prevent recurrence. The failure of resolution despite culture-directed intravenous meropenem therapy and percutaneous drainage highlights a key principle in abscess management: a persistent undrained source will often negate otherwise appropriate medical and percutaneous therapy, as was clearly demonstrated in the present case. Of note, no prior report has documented a non-Meckelian ileal diverticulum as the infectious origin, highlighting the distinctiveness of the present case.

Fusobacterium and colorectal disease

Although the causative species in this case was F. naviforme, particular attention has been paid to F. nucleatum, which is strongly associated with colorectal cancer. Mechanistically, F. nucleatum has been shown to contribute to carcinogenesis through adhesion molecules such as FadA and Fap2, and activation of the Wnt/β-catenin pathway [9,10]. However, unlike F. nucleatum, the direct link between F. naviforme and carcinogenesis remains unclear. Therefore, when Fusobacterium species are detected in liver abscesses, evaluation should include exclusion of not only inflammatory lesions such as diverticulitis and appendicitis but also colorectal neoplasia, although the strength of this association likely varies by species.

Clinical recommendations

・In cases where Fusobacterium-related liver abscess is suspected: optimize anaerobic cultures and employ molecular identification when necessary.

・Perform contrast-enhanced CT including the lower abdomen, and if colorectal pathology is suspected, proceed with colonoscopy.

・When conservative management fails or imaging suggests a persistent source, early surgical intervention should be considered.

These steps may improve early recognition and optimize outcomes in this rare but clinically important condition.

## Conclusions

This report emphasizes the importance of considering rare pathogens and unusual gastrointestinal sources in patients with persistent PLA. When Fusobacterium species are isolated, clinicians should expand the diagnostic evaluation beyond the hepatobiliary system and dental disease to include lower gastrointestinal pathology. Small abscesses may respond to antibiotics, but larger or refractory cases require invasive management, including drainage and, when indicated, surgical resection for definitive source control. To our knowledge, this is the first report of a F. naviforme liver abscess caused by a non-Meckelian ileal diverticulum. Recognition of such atypical sources is essential, as conservative treatment alone is often insufficient. Early surgical intervention, guided by careful imaging and microbiological findings, can result in rapid resolution and favorable outcomes. These insights may help optimize management strategies for similarly challenging cases in the future.
